# Dimensional accuracy and precision and surgeon perception of additively manufactured bone models: effect of manufacturing technology and part orientation

**DOI:** 10.1186/s41205-024-00203-4

**Published:** 2024-02-20

**Authors:** Emir Benca, Barbara Eckhart, Alexander Stoegner, Ewald Unger, Martin Bittner-Frank, Andreas Strassl, Claudia Gahleitner, Lena Hirtler, Reinhard Windhager, Gerhard M. Hobusch, Francesco Moscato

**Affiliations:** 1 Department of Orthopedics and Trauma Surgery, Medical University of Vienna, Währinger Gürtel 18-20, Vienna, 1090 Austria; 2 Center for Medical Physics and Biomedical Engineering, Medical University of Vienna, Vienna, Austria; 3 Department of Biomedical Imaging and Image-guided Therapy, Medical University of Vienna, Vienna, Austria; 4 Center for Anatomy and Cell Biology, Medical University of Vienna, Vienna, Austria; 5 Ludwig Boltzmann Institute for Cardiovascular Research, Vienna, Austria; 6 Austrian Cluster for Tissue Regeneration, Vienna, Austria

**Keywords:** Additive manufacturing, Anatomical model, Bone, Surgery, Accuracy

## Abstract

**Background:**

Additively manufactured (AM) anatomical bone models are primarily utilized for training and preoperative planning purposes. As such, they must meet stringent requirements, with dimensional accuracy being of utmost importance. This study aimed to evaluate the precision and accuracy of anatomical bone models manufactured using three different AM technologies: digital light processing (DLP), fused deposition modeling (FDM), and PolyJetting (PJ), built in three different part orientations. Additionally, the study sought to assess surgeons’ perceptions of how well these models mimic real bones in simulated osteosynthesis.

**Methods:**

Computer-aided design (CAD) models of six human radii were generated from computed tomography (CT) imaging data. Anatomical models were then manufactured using the three aforementioned technologies and in three different part orientations. The surfaces of all models were 3D-scanned and compared with the original CAD models. Furthermore, an anatomical model of a proximal femur including a metastatic lesion was manufactured using the three technologies, followed by (mock) osteosynthesis performed by six surgeons on each type of model. The surgeons’ perceptions of the quality and haptic properties of each model were assessed using a questionnaire.

**Results:**

The mean dimensional deviations from the original CAD model ranged between 0.00 and 0.13 mm with maximal inaccuracies < 1 mm for all models. In surgical simulation, PJ models achieved the highest total score on a 5-point Likert scale ranging from 1 to 5 (with 1 and 5 representing the lowest and highest level of agreement, respectively), (3.74 ± 0.99) in the surgeons’ perception assessment, followed by DLP (3.41 ± 0.99) and FDM (2.43 ± 1.02). Notably, FDM was perceived as unsuitable for surgical simulation, as the material melted during drilling and sawing.

**Conclusions:**

In conclusion, the choice of technology and part orientation significantly influenced the accuracy and precision of additively manufactured bone models. However, all anatomical models showed satisfying accuracies and precisions, independent of the AM technology or part orientation. The anatomical and functional performance of FDM models was rated by surgeons as poor.

## Background

Additive manufacturing (AM), encompasses a range of technologies used to create 3D objects. In orthopedics, AM plays a crucial role in the production of anatomical models [1], surgical guides [2], instruments [3, 4], and implants [5]. Among its numerous advantages, the use of anatomical models has been reported to offer benefits such as direct visualization of malformations, enhanced anticipation of anatomical complexities, reduced operating time, improved time-efficiency, lower risks and complications, and decreased radiation exposure to patients [6]. Utilizing anatomical bone models enhances the conditions for strategizing and simulating intricate surgical procedures. These models provide surgeons with a tactile understanding of complex anatomical structures during surgical planning and practice. Furthermore, they offer the added benefit of allowing surgeons to practice using conventional surgical instruments, facilitating realistic discussions and rehearsals of various surgical techniques [7].

To manufacture an anatomical model, the case-specific anatomy is typically assessed using radiological imaging. The imaging data is then segmented to isolate the desired region of interest. This resulting 3D volume can undergo various post-processing steps before itis transferred to the AM machine’s workstation, where the operator can adjust its orientation during the manufacturing process. Part orientation describes the rotation of the part in the build space around the axes of the machine’s coordinate system [8]. Proper part orientation is essential in AM, as it significantly influences manufacturing time, the number of parts that can be produced simultaneously, the amount of build and support material required, and associated time and costs.

It is important to note that any settings during imaging, image segmentation, model design, and manufacturing process can introduce deviations from the true dimensions, which can affect the clinical treatment plan relying on the accuracy of the 3D model. A systematic review of AM’s performance in surgical applications, including 158 studies, revealed that unsatisfactory accuracy was the most prominent disadvantage, reported in 31 studies (21%) [6].

So far, there has been limited effort to explore the precision of the manufacturing process as well as the visual and haptic perception of bone models. Therefore, this study aims to evaluate the precision and accuracy of anatomical bone models manufactured using three different AM technologies: digital light provessing (DLP), fusion deposition modeling (FDM), and multijetting/PolyJetting (PJ), in three different orientations. Additionally, it seeks to assess surgeons’ perceptions of how well these models mimic real bone.

## Methods

**Fig. 1:**
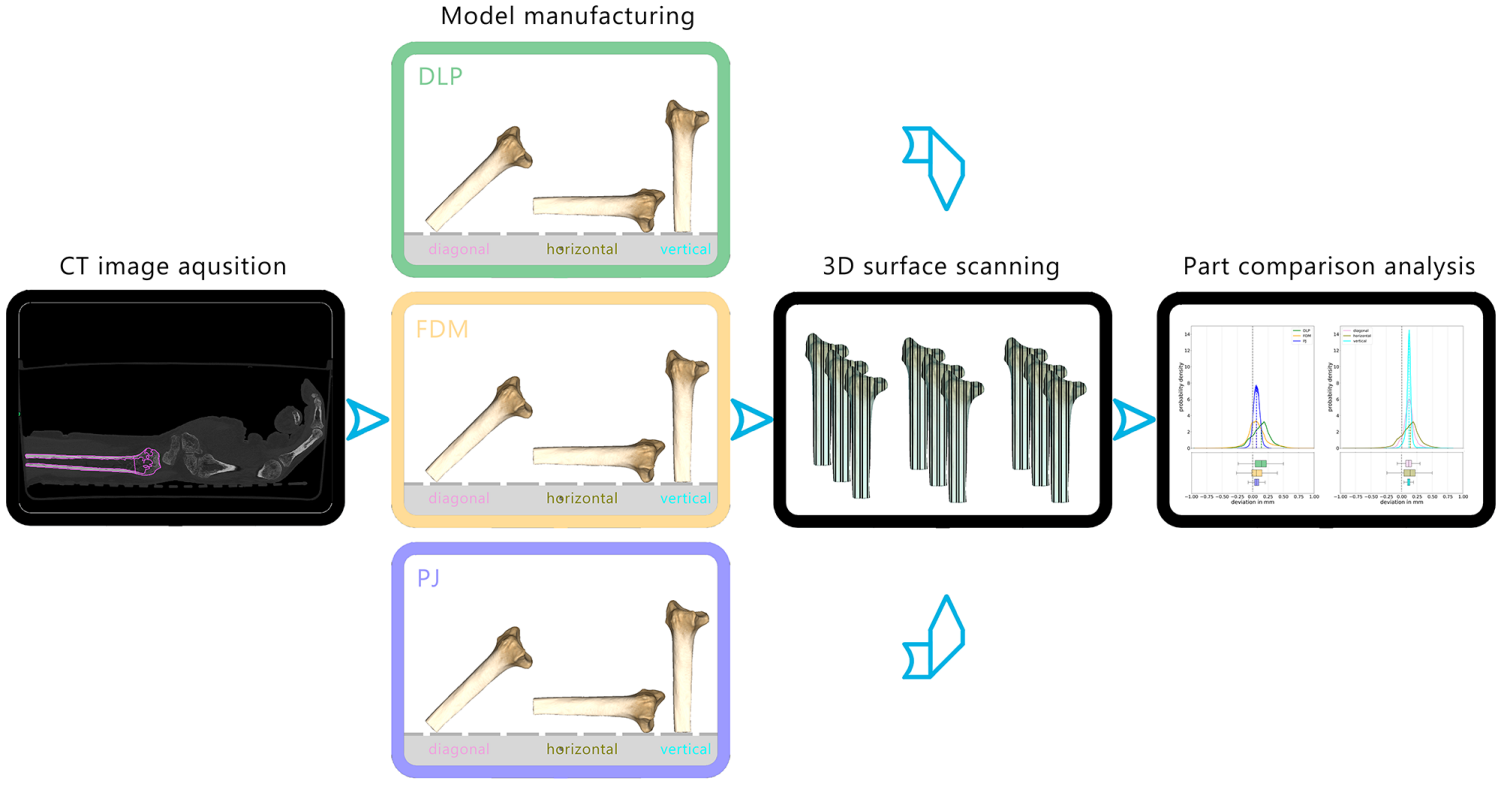
Methodology overview for dimensional accuracy and precision assessment: Six anatomical forearm specimens were scanned using a clinical CT scanner. These CT images were used as a basis for creating 3D CAD models of the radius, which served as the dimensional ground truth. Physical models were produced using three different additive manufacturing technologies: Digital Light Processing (DLP), Fused Deposition Modelling (FDM), and PolyJetting (PJ). Additionally, models were manufactured in three different part orientations, defined by the position of the radius axis relative to the build plate. These orientations included diagonal, horizontal, and vertical positioning. The surfaces of all manufactured models were then 3D-surface-scanned. The 3D-scanned data were compared with the original CAD models derived from the CT data. Data analysis was conducted to assess the impact of different technologies and part orientations on the dimensional accuracy and precision of the additively manufactured models

**Fig. 2:**
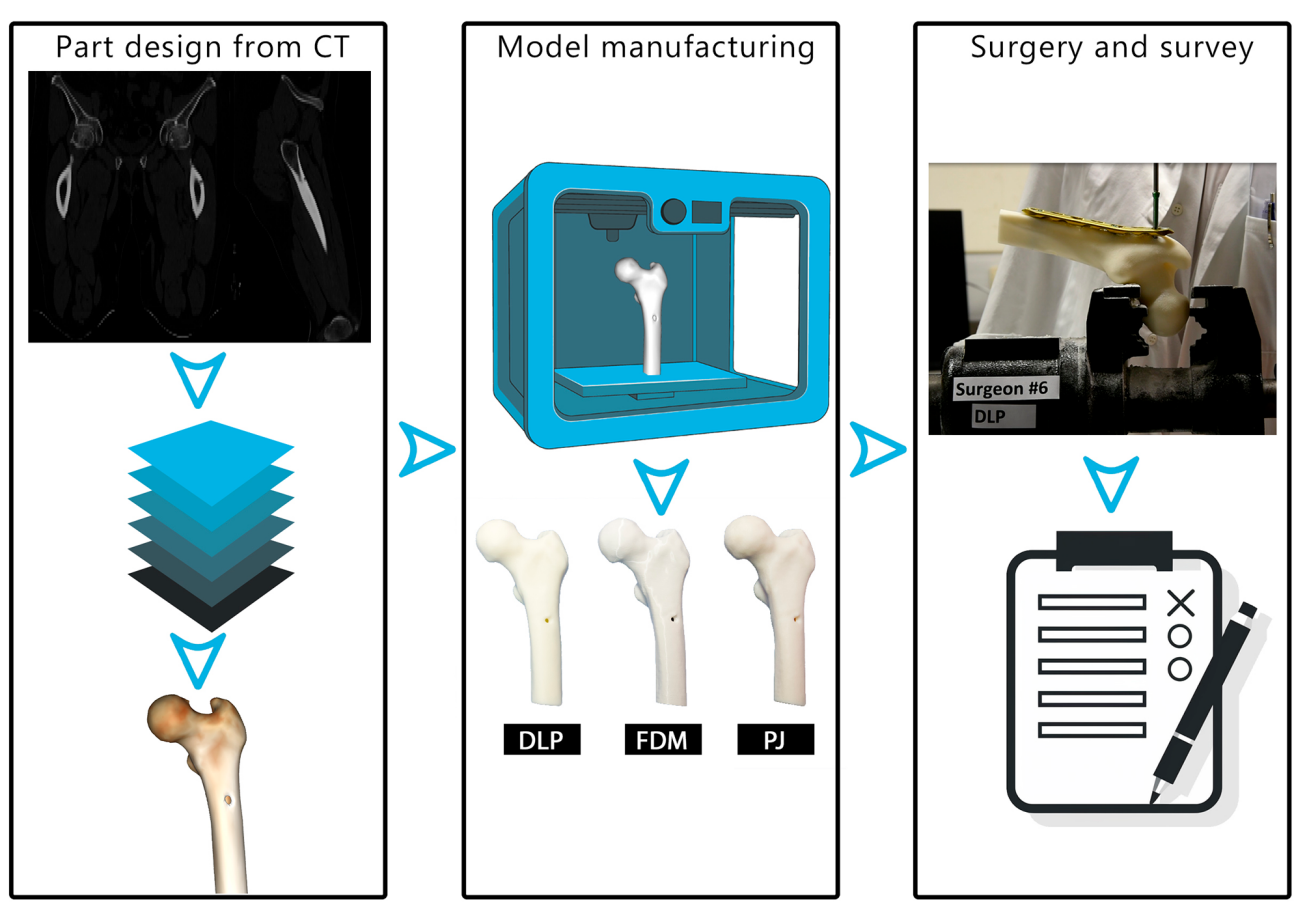
Methodology overview for surgeons’ perception assessment: CT imaging data from a patient with metastasis in the left proximal femur was used to create a CAD model. Anatomical models were manufactured using three different technologies: Digital Light Processing (DLP), Fused Deposition Modelling (FDM), and PolyJetting (PJ). Six surgeons conducted osteosynthesis procedures on anatomical models from all three technology groups. Surgeons’ performance and perception were assessed through a questionnaire that inquired about their experiences with each model

The study comprised two parts. In the first part (Fig. 1) computer-aided design (CAD) models derived from CT scans of forearm specimens were utilized to manufacture corresponding distal radius models using different additive manufacturing (AM) technologies and part orientations. The primary objective was to conduct a direct comparison between the manufactured models and their true dimensions, assessed from surface scans of the corresponding bone specimens. This comparison allowed to quantify the impact of AM technology and part orientation on dimensional accuracy.In the second part (Fig. 2), surgeons conducted an osteosynthesis on bone models manufactured using different AM technologies. Subsequently, their evaluation of each model’s performance was assessed through a questionnaire.

### Specimens

Six anatomical forearm specimens were collected for this study, consisting of two left forearms and four right forearms (four paired, two unpaired). These specimens included two left forearms and four right forearms, with four of them being paired and two unpaired. The specimens were obtained from a total of four body donors, comprising three females and one male, with ages ranging from 62 to 80 years. Prior to their passing, these donors had granted written consent to the Center for Anatomy and Cell Biology for the utilization of their bodies in research and educational purposes. The study obtained approval from the Ethics Committee of the Medical University of Vienna (Nr. 2003/2019). The specimens were part of a larger population utilized to evaluate the accuracy of additively manufactured models and their features concerning radiological imaging and image segmentation [9]. The radius models were segmented out of CT scans of anatomical forearm specimens. CT scans were performed using a 3rd generation dual-source CT scanner (SOMATOM Force; Siemens Healthineers AG, Forchheim, Germany) equipped with a comb filter enabling high-resolution imaging. Following settings were used: 120 kV tube voltage, 300 mAs tube current-time-product, 64 × 0.6 mm collimation, 1 s rotation time, and 0.35 pitch. For image reconstruction 512 × 512 matrix size, 0.4 mm slice thickness, 0.4 mm slice increment, and Ur77u\3 reconstruction kernel was used. The image field of view was limited to the size of a single specimen.

### Image segmentation

Image processing was conducted using Mimics Research (V21.0, Materialise NV, Leuven, Belgium). In all CT image series, segmentation was performed by applying a lower threshold between the cortex and soft tissue of the diaphysis using a multi-manual approach. There was no limitation on the upper threshold. Radii were then separated from other bones using the ‘region grow’ tool. CAD parts were generated with the ‘optimal’ setting, and post-processing was carried out using the ‘wrap’ tool (with ‘smallest detail’ set to one pixel and ‘gap closing distance’ set to half a pixel) and the ‘smooth’ tool (two iterations, smooth factor 0.3, following the methodology described in [10]).

### Manufacturing technologies

Each radius model was produced using three different AM technologies (see Table 1 for detailed machine and manufacturing process information). These models were manufactured in three distinct part orientations: with the bone axis aligned parallel to the build plate (horizontal), orthogonal to it (vertical), and diagonal to it. Due to the low thickness of the cortical bone shell, frequent damage occurred in the radii models during manufacturing. To address this issue, the models were manufactured with a consistent wall thickness of 2.5 mm, and the trabecular bone was omitted.

**Table 1:** Descriptive information on the machines, materials and process (manufacturing of a proximal femur model) details for each AM technology

	Digital Light Processing (DLP)	Fused Deposition Modelling (FDM)	PolyJet (PJ)
Machine	MARS 3 (and Mercury Plus wash and curing station)	Prusa MINI	Objet500 Connex3
Technology	masked stereolithography	material extrusion	material jetting
Build area (mm)	143 × 90 × 175	180 × 180 × 180	490 × 390 × 200
Resolution (X/Y/Z axis) (dpi)	600	600	600/600/1600
Accuracy (µm)	35	≤ 300	200
Estimated purchase price (EUR)	400	500	150,000
Licensed for medical use	no	no	yes
Used material (commercial name)	photopolymer (ELEGOO Water washable photopolymer resin in white)	thermoplastic (Prusament PETG in signal white)	photopolymer (VeroPureWhite) and support (SUP706 B)
Material costs per radius model (EUR)	0.54/0.37/0.48 in horizontal/vertical/diagonal orientation	0.74/0.66/0.81 in horizontal/vertical/diagonal orientation	8.27/12.27/18.58 in horizontal/vertical/diagonal orientation
Material costs per femur model (EUR)	6.36	7.83	66.63
Manufacturing duration for radius model (hr:min)	02:20/05:40/06:22 (42:00 for 18 models)	03:25/03:10/04:20 (62:16 for 18 models)	01:56/06:48/07:43 (16:21/27:00/43:31 for six models) in horizontal/vertical/diagonal orientation
Manufacturing duration femur model (hr:min)	10:02	13:37	7:30
Major post-processing steps	Removing support Washing model Letting model dry Curing model Cleaning machine	Removing support structures by hand	Removing support material with water Washing in 2% NaOH
Estimated post-processing duration per model (min)	25	< 5	30
Estimated total duration per femur model (hr:min)	10:30	13:40	8:00
Waste	isopropyl alcohol	thermoplastic support structures	support material

### 3D surface scanning

After the manufacturing process, the surfaces of all 3D distal radii models were digitized using a high-resolution 3D surface scanner (SmartSCAN HE-C8; Hexagon AB, Stockholm, Sweden). This scanner features a 210 mm x 160 mm field of view and offers a 14 μm feature accuracy. Each model was securely positioned on a tilt and rotation table (Turn-Tilt Unit M; Hexagon AB, Stockholm, Sweden) and underwent scanning while being rotated around all three axes. Combining fringe projection and stereometry, the scanner provided surface information of the object in the STL file format.

### Part comparison analysis

To assess dimensional differences between the manufactured models and the corresponding CAD models generated from CT imaging data, part comparison analyses were performed in Materialise 3-Matic Research 17.0 (Materialise NV, Leuven, Belgium). Initially, artefacts from 3D surface scan were removed using the manual trimming tool. The corresponding CAD model was then imported and registered into the 3D model generated from surface scans using the N-point registration tool. In each model, three anatomical landmarks (the styloid process, the dorsal tubercle (Lister’s tubercle) and the most proximal point of the medial border) were selected as registration points. This allowed for an automatic superimposition of the two models.

Further refinement of the registration was carried out using the global registration tool, with specified parameters (distance threshold: 1.000, 20 iterations, sample percentage 100). This refinement was performed twice or thrice, as needed. We employed the software’s part comparison analysis tool to calculate the dimensional deviation between the surface points of the two models, resulting in a signed analysis. The density distribution histograms were exported as text files and then analyzed them using a custom Python script in Spyder (version 4.2.5, Python 3.8, The Scientific Python Development Environment). This analysis involved computing various statistical measures, including the minimum (MIN), maximum (MAX), median, interquartile range (IQR), mean, standard deviation (SD), and root mean square error (RMSE). Furthermore, density distribution histograms, probability density functions (pdf, calculated using a kernel-density estimate with Gaussian kernels), and boxplots were plotted for data visualization.

### Surgical simulation

For the purposes of this study, a clinical case involving a 72-year-old female patient with thyroid cancer and a lytic metastasis in the left proximal femur was selected retrospectively. To obtain the necessary data, including the patient’s medical records, operative report, and radiological imaging, we retrieved the relevant information from the clinical database. The utilization of retrospective clinical data was approved by a separate approval by the Ethics Committee of the Medical University of Vienna (Nr. 1284/2019). In this case, the metastasis was surgically removed and bone stabilization was achieved using a locking compression plate (LCP) (4.5/5.0 LCP Plate (426.571 S), Synthes GmbH, Oberdorf, Switzerland) along with seven 5.0 mm self-tapping locking screws (Synthes GmbH, Oberdorf, Switzerland). The screws used had lengths of 32 mm (x 2), 36 mm, 38 mm, 40 mm, 50 mm, and 60 mm.

To evaluate the visual and haptic perception of bone models manufactured with the three AM technologies by surgeons, the femur from the mentioned clinical case was manufactured six times using each of the three AM technologies. The part orientation was automatically selected by the device’s software and was not altered by the operator. A group of six surgeons with varying levels of experience then replicated the same surgical procedure as described earlier.

**Fig. 3:**
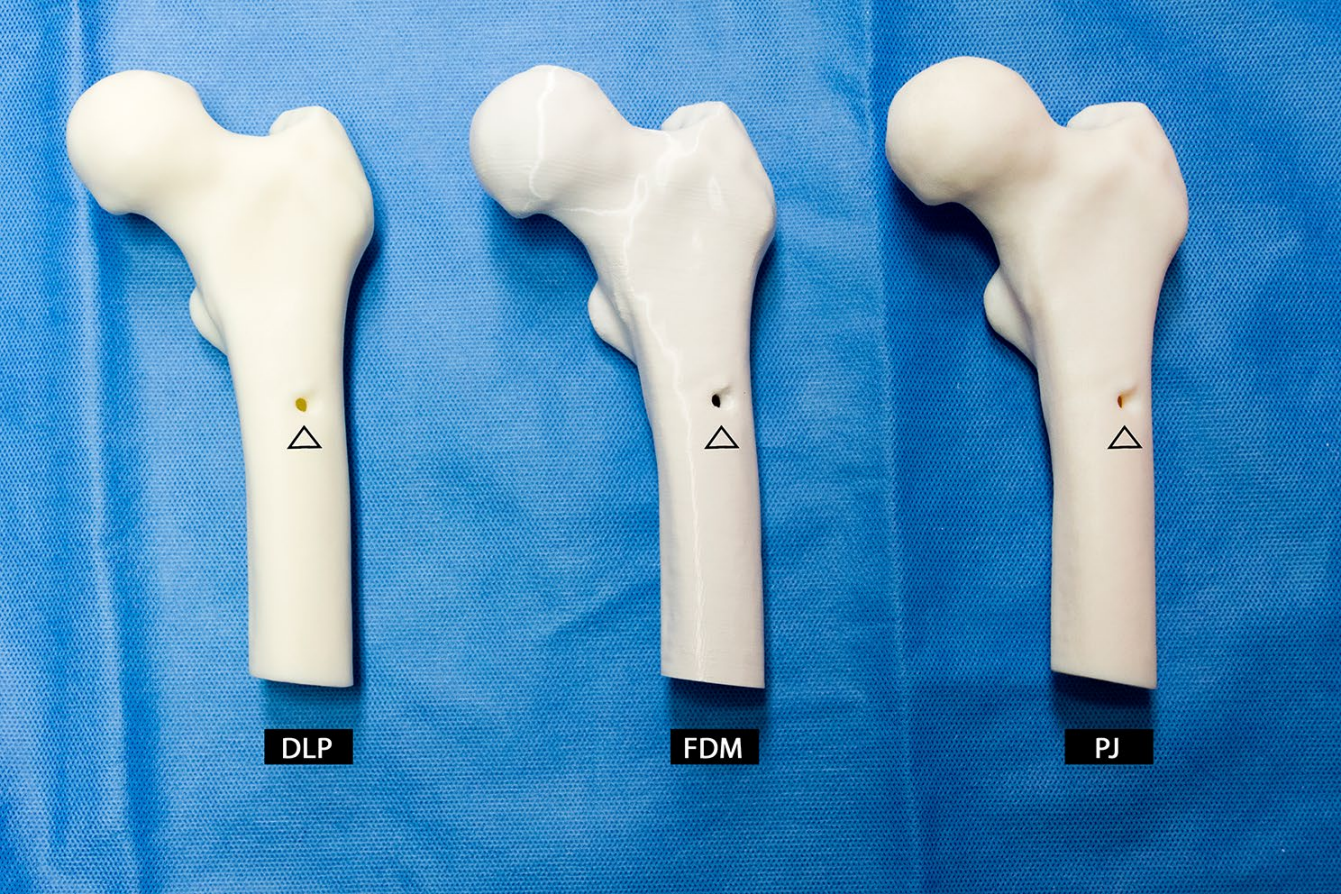
Additively-manufactured left proximal femur models with metastasis (as indicated) from CT data of 72-year-old female patient with thyroid cancer. Models were manufactured with a digital light processing (DLP), a fused deposition modelling (FDM), and a PolyJetting (PJ) machine

For CAD model design, the DICOM data were segmented using a manual threshold within the range of 200 to 3071 Hounsfield Units (HU). Due to the relatively coarse CT image slice thickness of 4.0 mm, extensive manual post-processing was required. The fine structure of the cancellous bone tissue was manually filled in entirely. Since this study focused on the visual and haptic aspects of the anatomical models, the emphasis was placed on achieving a smooth surface and clear visibility of the lesion (Fig. 3). Manual editing of individual slices ensured the accurate representation of the metastasis, which was presented in the CAD model by entirely removing tissue in the affected region. The CAD femur model was finalized and cropped to fit the maximum building volume in Materialise 3-Matic Research. In a preliminary experiment, which was limited to model drilling, it was determined that the FDM models would be manufactured with 100% infill, as lower infill densities were considered too soft.

For the surgical procedure, the femoral head was securely secured in a mechanical vise to ensure stability. Prior to the surgery, each surgeon received a briefing about the study’s objectives and was provided access to intra- and post-operative radiographs of the clinical case. They were also familiarized with the instruments, implants, and their respective sizes. The selection of implants was based on the operative report, and the LCP plate was initially pre-shaped to match the anatomical requirements by an experienced surgeon (GMH). Each surgeon then positioned the plate on the bone and secured it in place using a screw clamp. For each LCP hole, a 4.3 mm LCP drill sleeve was inserted, followed by drilling a 4.3 mm screw hole and inserting the specific screw using a power tool. Each surgeon performed the three procedures one after the other, selecting 3D models in randomized order. To gain further insight into the haptic feedback of the models, all screws were removed, and an osteotomy of the bone shaft was performed with an oscillating saw. Each simulation was documented on video. Following each simulated surgical procedure, the surgeon was asked to provide ratings for the specific 3D model based on a set of ten questions.

### Questionnaire

A questionnaire comprising 10 closed-scale questions was designed to assess surgeons’ perception of the quality and haptic property for each 3D model following simulated surgical procedure (Table 2). The questions were categorized into three groups: [11] three questions related to anatomy and pathology, allowing reference to preoperative CT data [1], six surgery-related questions, categorized by anatomical location (e.g., metaphysis, diaphysis), and [2] one question regarding the potential use of the model in clinical preoperative planning. The content of the questionnaire was based on similar studies comparing the quality and haptic properties of 3D-printed bone models, as identified during a literature search [12, 13]. Respondents were asked to indicate their level of agreement or disagreement with a series of statements. Responses were recorded using a 5-point Likert scale, ranging from 1 to 5. A rating of 1 represented the lowest level of agreement or satisfaction (e.g., ‘Strongly Disagree’), while a rating of 5 indicated the highest level of agreement or satisfaction (e.g., ‘Strongly Agree’).

**Table 2:** Questionnaire using 5-point Likert scale to assess surgeons’ perception after performing a simulated surgery on each of the additively-manufactured 3D femur models

Question No.	Question	Location
**Anatomical performance**		
1	The 3D model feels like a real bone to touch.	n/a
2	The 3D model resembles bone in colour.	n/a
3	The metastatic lesion is realistically represented in the 3D model compared to the radiological images.	n/a
**Functional performance**		
4a 4b	The tactile perception and resistance during the drilling of the 3D model resemble that of bone.	diaphysis
		metaphysis
5a 5b	The transition from cortical bone to medullary cavity is observed during drilling.	diaphysis
		metaphysis
6a 6b	The tactile perception and resistance during screw placement resembles that of bone.	diaphysis
		metaphysis
7a 7b	The tactile perception and resistance during screw removal resemble that of real bone.	diaphysis
		metaphysis
8a 8b	The tactile perception and resistance during plate placement resemble that of bone.	diaphysis
		metaphysis
9	The tactile perception and resistance during osteotomy resemble that of bone.	diaphysis
**Overall clinical value**		
10	Such 3D models would be useful for preoperative planning and implant selection.	n/a

### Statistical analysis

Statistical analysis related to model accuracy and precision was performed utilizing SciPy [14]. The Shapiro-Wilk test revealed that the majority of the data did not follow a normal distribution. Consequently, the Kruskal-Wallis one-way analysis of variance was employed to assess differences between different technologies for the specific part orientation, and between different part orientations for the specific technology. Data from all six samples were pooled and evaluated for either the additive manufacturing technology or the part orientation. In the case of a significant Kruskal–Wallis ANOVA, the Dunn’s test with Bonferroni correction was performed pairwise between specific pooled groups using the scikit-posthoc Python package.

For the analysis of the questionnaire data, IBM SPSS Statistics 25 (IBM, Armonk, NY, USA) was utilized. The questions were categorized into three distinct categories: anatomical performance (questions 1–3), functional performance (questions 4a-9), and overall clinical value (question 10) of the anatomical models. The Shapiro-Wilk test indicated that most data were not normally distributed. Hence, the Kruskal-Wallis one-way analysis of variance was employed to examine differences in scores between different technologies for each question category. If a significant Kruskal-Wallis test was observed, the Dunn’s test with Bonferroni correction was performed pairwise between specific additive manufacturing technologies.

An α level of < 0.05 was set for all statistical tests.

## Results

### Dimensional accuracy and precision

While it was feasible to manufacture all bone models, manufacturing problems occurred in all technologies. Reproducing small wall thickness in radii models utilizing DLP was not feasible as it often fragmented. Therefore, all models were manufactured (for all technologies) with a constant wall thickness of 2.5 mm. Also, a 4.0 mm drainage hole for the resin was designed in the distal shaft.

**Fig. 4:**
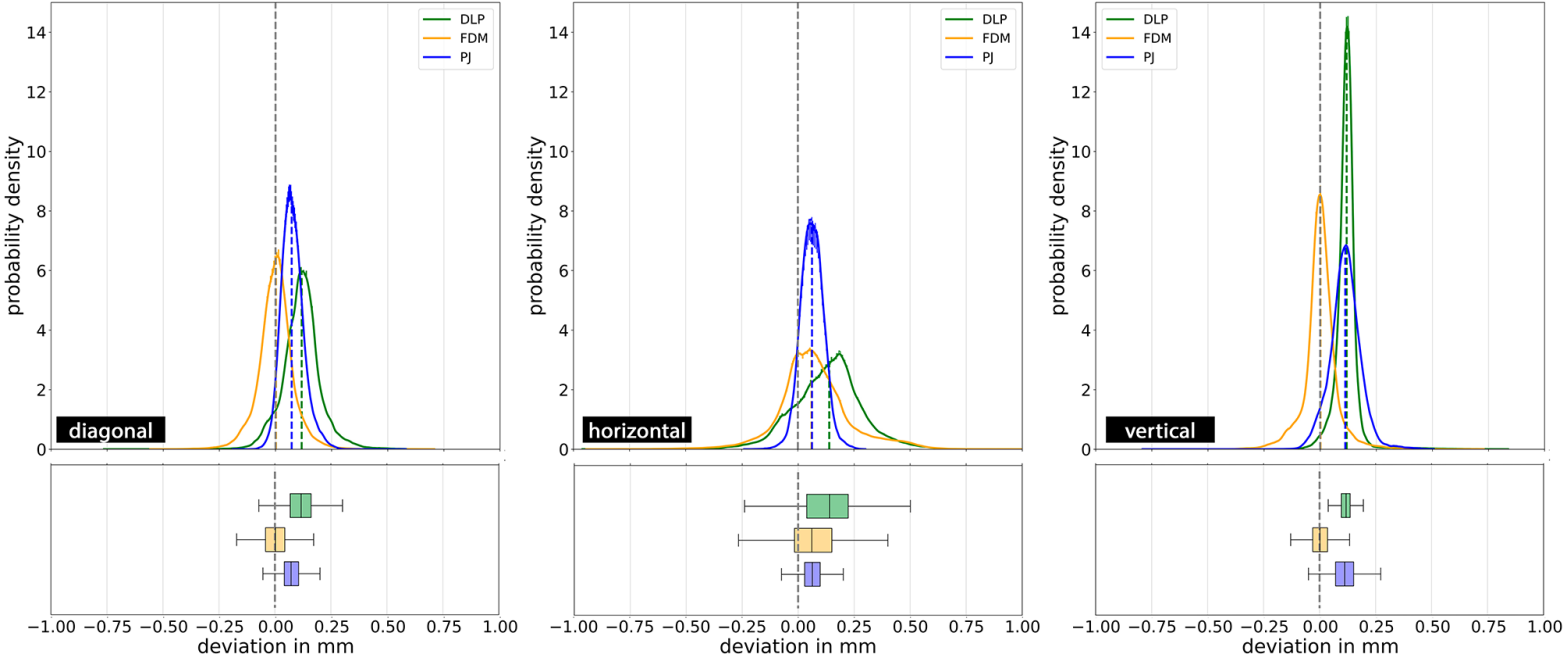
Effect of different part orientations on 3D model dimensions for the specific manufacturing technologies. Probability density functions are shown with histograms and corresponding boxplots (due to a large number of data points and for better visibility, outliers are not plotted (∼300,000 outliers)). Abbreviations: DLP: Digital Light Processing; FDM: Fused Deposition Modelling; PJ: PolyJetting

**Fig. 5:**
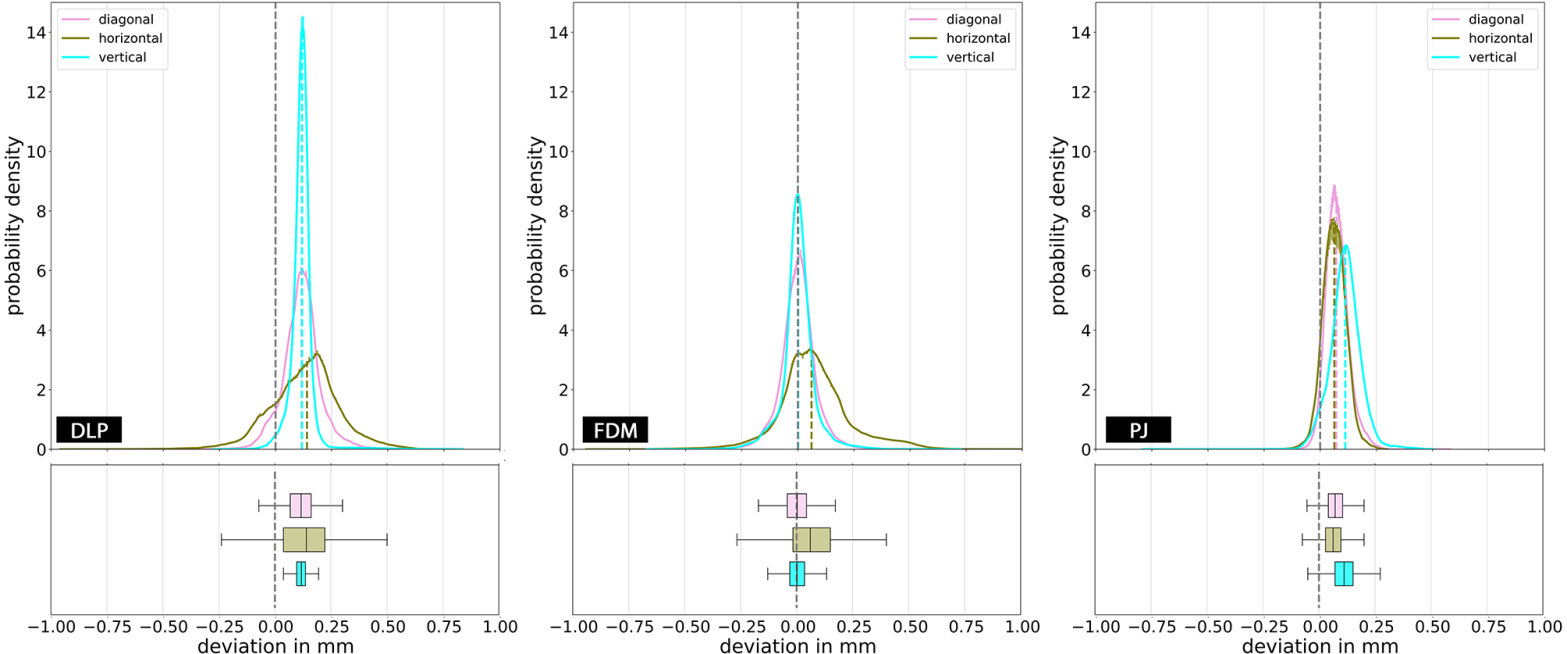
Effect of different manufacturing technologies on 3D model dimensions for the specific part orientations. Probability density functions are shown with histograms and corresponding boxplots (due to a large number of data points and for better visibility, outliers are not plotted (∼300,000 outliers)). Abbreviations: DLP: Digital Light Processing; FDM: Fused Deposition Modelling; PJ: PolyJetting

Deviations of 3D model dimensions for different AM technologies and (part) orientations is summarized in Table 3 and visualised in Figs. 4 and 5.

**Table 3:** Descriptive outcome measures for the deviation of 3D model dimensions (mm) for different AM technologies and (part) orientations

Technology	Orientation	Mean	SD	RMSE	MIN	MAX	Median	IQR
DLP	diagonal	0.12	0.09	0.14	-0.76	0.69	0.12	0.09
DLP	horizontal	0.13	0.16	0.20	-0.96	0.75	0.14	0.19
DLP	vertical	0.12	0.05	0.13	-0.28	0.84	0.12	0.04
FDM	diagonal	0.00	0.08	0.08	-0.56	0.71	0.00	0.09
FDM	horizontal	0.07	0.16	0.18	-0.94	1.04	0.06	0.17
FDM	vertical	0.00	0.07	0.07	-0.68	0.73	0.00	0.07
PJ	diagonal	0.08	0.05	0.09	-0.19	0.58	0.07	0.06
PJ	horizontal	0.06	0.05	0.08	-0.24	0.30	0.06	0.07
PJ	vertical	0.11	0.07	0.13	-0.79	0.51	0.11	0.08

Abbreviations: SD: standard deviation, RMSE: root mean squared error, MIN: minimum, MAX: maximum, IQR: interquartile range

In general, considering the substantial number of data points within each group (mean: 3.9 × 10^6^), every statistical analysis resulted in statistically significant differences (*p* < 0.001) between additive manufacturing technologies and part orientations in all instances.

### Surgeons’ perception

**Fig. 6:**
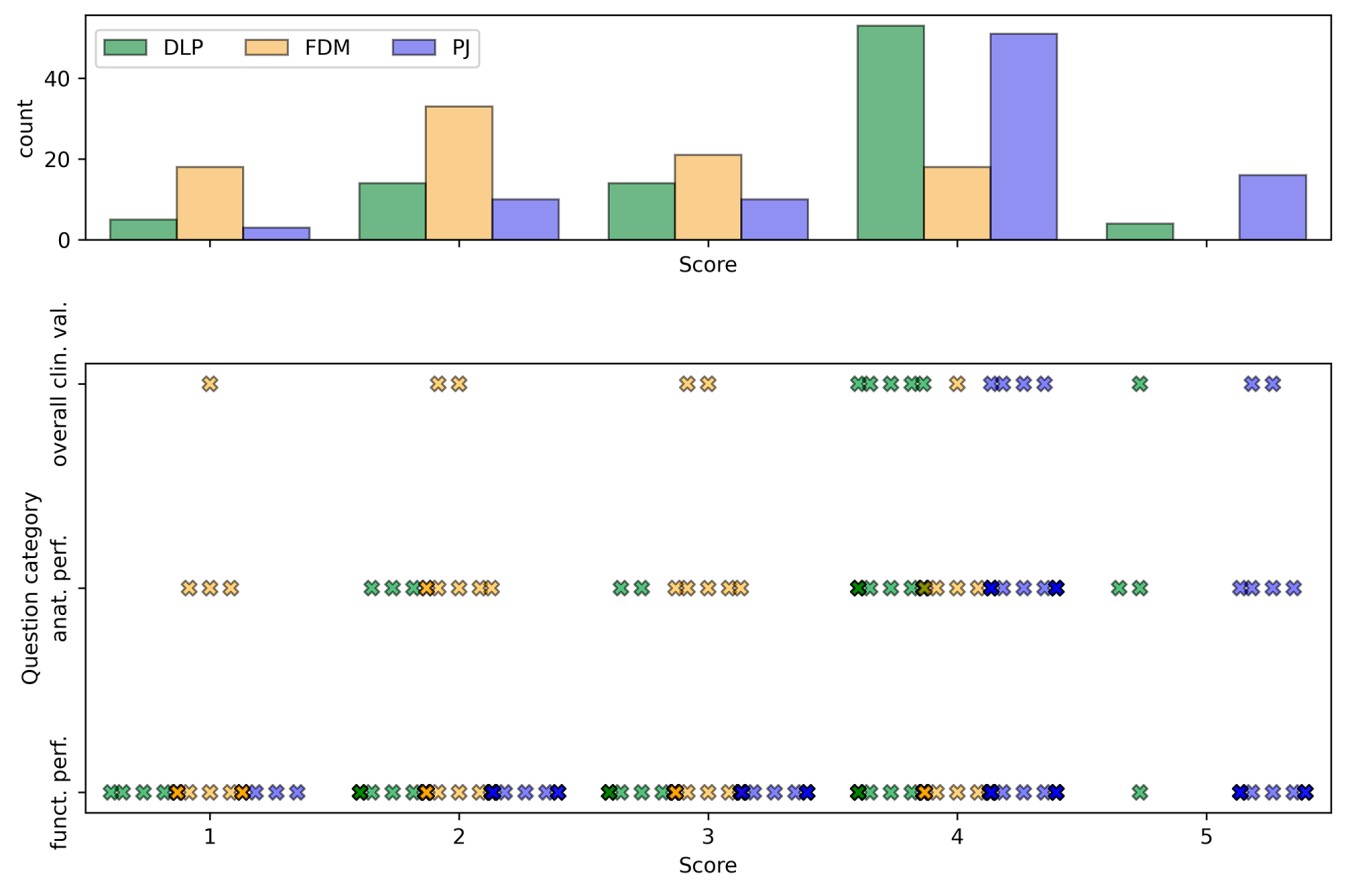
Total score count (top) and score distribution for each question group (bottom) and technology. The scores, ranging from 1 (strong disagreement) to 5 (strong agreement), were provided by six surgeons following simulated surgery

**Fig. 7:**
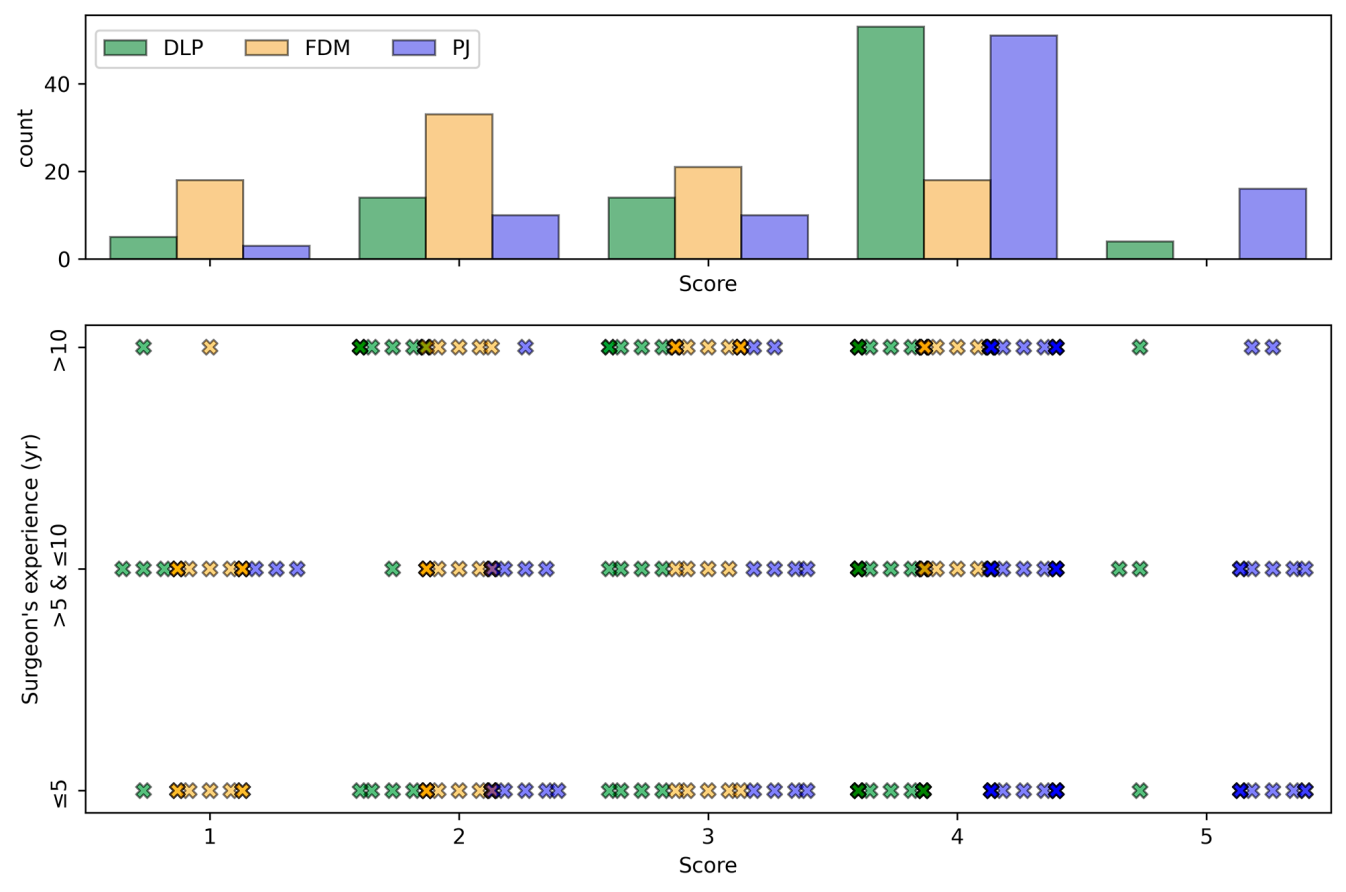
Total score count (top) and score distribution for defined surgeons’ experience levels (bottom) and each technology. The scores, ranging from 1 (strong disagreement) to 5 (strong agreement), were provided by six surgeons following simulated surgery

The theoretical maximum score sum for 15 questions and 6 respondents amounted to 450, resulting in a normalized percentage value of 100%. Notably, PJ received the highest score sum (337 or 74.9%), followed by DLP (307 or 68.2%), and FDM (219 or 48.7%). The mean scores for each question category, location, AM technology, and surgeons’ levels of experience are presented in Table 4 and visually depicted in Figs. 6 and 7.

**Table 4:** Descriptive results of the questionnaire analysis, categorized by question group and in total. Scores are presented as mean ± SD (standard deviation) for surgeons with varying levels of experience (2 surgeons per experience group) expressed in years (yrs) from the commencement of their trauma and orthopedic surgery residency, as well as for all surgeons collectively

Question category (n)	Location	3D printing technology	Score			
			≤ 5 yrs	> 5 & ≤ 10 yrs	> 10 yrs	total
Anatomical performance (3)	n/a	DLP	3.50 ± 0.84	4.17 ± 0.75	3.33 ± 1.03	3.67 ± 0.90
		FDM	2.33 ± 0.82	1.83 ± 0.75	3.50 ± 0.84	2.56 ± 1.04
		PJ	4.33 ± 0.52	4.33 ± 0.52	4.00 ± 0.00	4.22 ± 0.34
Functional performance (11)	metaphysis (5)	DLP	3.10 ± 1.04	2.60 ± 1.20	2.70 ± 1.00	2.80 ± 1.11
		FDM	1.80 ± 0.60	1.80 ± 1.17	3.20 ± 0.75	2.27 ± 1.09
		PJ	2.90 ± 0.94	2.90 ± 1.30	3.70 ± 0.64	3.17 ± 1.07
	diaphysis (6)	DLP	3.75 ± 0.60	4.00 ± 0.00	3.25 ± 0.92	3.67 ± 0.71
		FDM	1.83 ± 0.55	2.75 ± 1.01	2.92 ± 0.86	2.50 ± 0.96
		PJ	4.17 ± 0.80	3.50 ± 1.26	4.00 ± 0.41	3.89 ± 0.94
Overall clinical value (1)	n/a	DLP	4.50 ± 0.71	4.00 ± 0.00	4.00 ± 0.00	4.17 ± 0.41
		FDM	2.00 ± 0.00	2.00 ± 1.41	3.50 ± 0.71	2.50 ± 1.05
		PJ	4.50 ± 0.71	4.00 ± 0.00	4.50 ± 0.71	4.33 ± 0.52
Total (15)	n/a	DLP	3.53 ± 0.90	3.57 ± 1.04	3.13 ± 1.01	3.41 ± 0.99
		FDM	1.93 ± 0.64	2.20 ± 1.12	3.17 ± 0.83	2.43 ± 1.02
		PJ	3.80 ± 1.03	3.50 ± 1.25	3.93 ± 0.52	3.74 ± 0.99

There were statistically significant differences between the technologies in simulated surgeries for their anatomical and functional performance (both *p* < 0.001), as well as for their clinical value (*p* = 0.005). Results from pairwise comparisons revealed that FDM models underperformed in all question categories compared to DLP and PJ models. The results are presented in Table 5.

**Table 5:** Results of the pairwise comparison (Dunn’s test with Bonferroni correction) for differences between scores for the specific additive manufacturing technologies for each question category

Anatomical performance			
	DLP	FDM	PJ
DLP	-	0.007	0.253
FDM	0.007	-	< 0.001
PJ	0.253	< 0.001	-
Functional performance			
	DLP	FDM	PJ
DLP	-	< 0.001	0.438
FDM	< 0.001	-	< 0.001
PJ	0.438	< 0.001	-
Overall clinical value			
	DLP	FDM	PJ
DLP	-	0.026	> 0.999
FDM	0.026	-	0.008
PJ	> 0.999	0.008	-

## Discussion

The objective of this study was to evaluate the dimensional accuracy and precision of anatomical bone models manufactured using various additive manufacturing (AM) technologies and part orientations, with a specific focus on their suitability for surgical simulation. The results underscored the significant impact of both the choice of technology and part orientation on the accuracy and precision of the models. However, when considering the overall mean root mean square error (RMSE) of 0.12 ± 0.04 mm and absolute extrema of ≤ 1 mm, it can be concluded that the impact is generally deemed negligible for most applications in orthopedics and trauma surgery. As a result, even the most basic technology exhibited commendable performance and can be regarded as a reliable option for manufacturing bone models. Surprisingly, models manufactured with a table-top FDM machine displayed the highest level of accuracy. Part orientations did not have a clinically relevant effect on the accuracy and precision of the manufactured models for any of the investigated technologies. However, the horizontal orientation did reduce the precision of the DLP- and FDM-manufactured models, allowing for global maxima in the range of 1 mm. Visual examination of the registered models revealed dimensional inaccuracies in contact regions of the models with the support structures. Support structures are typically hollow or cellular structures required as external support for overhanging part layers. For applications requiring reproducibility, it is advisable to fine-tune the support structure generation parameters, including contact distance, depth, width, density of supports, and angles of contact.

Even models with high accuracy may have limitations related to precision and the presence of outliers beyond the submillimeter range, which can affect their suitability for clinical use. For example, low precision in fracture surfaces could compromise their utility in fracture reduction procedures, and deteriorated surfaces of anatomical features like cavities or lesions could negatively impact surgical treatment decisions, including the selection of implant size and type. Hence, any contact of support structures with surfaces of clinical interest should be avoided or minimized. Part orientation is, therefore, an important aspect of the manufacturing process and it should be considered as a relevant quality feature [8]. Considerations should include model requirements (surface quality, dimensional accuracy), technical feasibility (machine build space, accessibility of support structures), sustainability (support volume, which often represents waste in the AM process), time efficiency (part manufacturing and finishing complexity), and cost-effectiveness (building costs) [15]. In the present study, surfaces were not further mechanically processed to remove residual support structures, as it it was not feasible to standardize this step and prevent alterations of the adjacent areas. PJ models exhbited the least deviation from the mean deviation for all part orientations, making PolyJetting the most reliable technology for clinical applications. However, it is important to note that the material costs of the PJ models were over 10 times higher than the material costs of the other two technologies. Additional costs for each technology, including the purchase price and equipment maintenance, need to be taken into account. This makes the PolyJet machine the least cost-effective and impractical for basic applications. At the same time, the manufacturing and post-processing of femur models took significantly less time when using PJ compared to DLP (which took 30% longer) or FDM (which took 70% longer). Finally, the impact of orientation also varies between technologies. On the one hand, varying part orientations in FDM has little effect on build duration due to the single printhead, which needs to trace the entire model’s volume, regardless of the orientation. Thus, only additional supports or raft layers significantly influence manufacturing duration. DLP machines show greater variations in manufacturing duration, as models are created layer by layer. A horizontal orientation will result in fewer layers in total, as each layer covers a larger cross-section. In contrast, vertical orientation will lead to much longer manufacturing durations. Both DLP and FDM tabletop machines, along with their maintenance, are relatively inexpensive, making them suitable for educational and training purposes.

The models manufactured using the PolyJet machine, Connex500, consistently exhibited slight enlargement compared to the CAD models, with mean discrepancies ranging from 0.08 to 0.11 mm. This inaccuracy can be attributed, at least to a certain extent, to the limited resolution of the machine, which becomes evident in the part’s contour. In the PolyJetting process, for each layer of the STL file, the machine’s nozzle moves along the theoretical exact contour of the CAD geometry, superimposing the centerline of the material beam that is jetted. Considering that the material beam has a finite width (42.3 μm for the Connex500 with a build resolution of 600 dpi in the x-y plane), the resulting theoretical error in the model’s contour can be as much as half the material beam diameter [16]. This edge contour is typically not well connected and can be effectively removed through mechanical means, such as polishing, ideally correcting this error. With the lowest RMSE (0.08 mm ≤ RMSE ≤ 0.13 mm) the PJ models were most precise among all AM technologies making them most reliable for clinical applications. It’s worth noting that the observed inaccuracies in all technologies may be partially attributed to methodological limitations. Mathematical representations of complex surfaces, like the triangulated representation used in this study, inherently involve certain approximations [17]. Additionally, calculating distances between two discretely represented surfaces lacks a perfect method. Given the high resolution of both segmentation and laser surface scans, assuming the distance between their two closest points is a valid approach for estimating the remaining distance between the surfaces.

Dimensional inaccuracies are attributed to a number of factors related to all processes including medical imaging [9, 18], image segmentation [9, 17, 19], model post-processing [9], geometry file conversion, part build [17], and finishing [15] (i.e., cleaning, polishing, etc.). It is important to stress that errors occurring in single processes or part layers propagate and transfer to the consecutive processes or layers. Thus, the dimensional inaccuracy of an anatomical model can be the result of accumulated errors [16]. Overall, the observed accuracies and precisions are consistent with that reported in the literature. Msallem et al. [20] conducted a study in which they replicated a single bony mandible ten times using five different AM technologies. In their research, all investigated machines exhibited errors of less than 0.5 mm. In another study [21], the accuracy of four distinct AM technologies was assessed by superimposing computer-aided design (CAD) models, generated from clinical CT data of anatomical bone samples, with the corresponding AM models. The results showed that the mean differences in surface geometry fell within the range of 0.1 to 0.2 mm for all the technologies. Additionally, Smith et al. [17] compared CAD models of anatomical hip and shoulder joints with the surface scans of corresponding models manufactured using an FDM machine. Their findings revealed an accuracy of 0.1 mm.

Due to the limited number of participating surgeons with varying levels of experience, the reported statistical analysis relied on a descriptive presentation of the survey data, limiting the extent to which valid conclusions can be drawn. The largest discrepancies were observed between the FDM and DLP as well as between the FDM and PJ models for the detriment of the FDM models for nearly all question categories, anatomical sites, and experience levels. Several factors unique to FDM technology contributed to this discrepancy. First, FDM models were manufactured using a PETG filament, a thermoplastic material with a low heat deflection and melting temperature. The heat generated during drilling and sawing, coupled with the limited heat conductivity of plastic, led to elevated temperatures at the contact points with the drill bit and surgical saw blade. Consequently, the material did not chip away but instead softened and melted, adhering to the drill bit and saw blade, causing them to jam. Furthermore, the FDM-manufactured models exhibited noticeable layer lines that were both visible and perceptible to touch. The surface quality of FDM models can be enhanced through post-processing techniques involving either mechanical or chemical methods, although these processes necessitate additional resources.

The functional performance of all models received lower ratings in the metaphyseal compared to the diaphyseal site. Participating surgeons reported that they did not encounter the anticipated two-phase drilling resistance typically experienced when drilling bicortically. The femur models were manufactured with 100% infill in the trabecular bone region to achieve a higher and more realistic drilling resistance. Previous research has shown that FDM-manufactured femur models tend to underestimate the stiffness and ultimate load of real bones [22]. As compressive strength is directly related to drilling resistance [23] replicating the drilling resistance of real bones in polymer models is not feasible. Thus, a trade-off between morphology and mechanical behavior must be considered, taking into account the specific purpose of the models. Also, it’s important to acknowledge that trabecular bone cannot be precisely replicated using most clinical imaging technologies and can be represented bya generic porous structure, such as the gyroid pattern [24].

During drilling, some DLP and PJ models experienced blowout, with flat fragments being expelled at the drill bit exit holes. This phenomenon is more likely to occur when using dull drill bits. In our study, a new drill bit was employed, and blowouts were observed as a drawback of the used materials. This aspect has a limiting potential for certain clinical applications and should, accordingly, be addressed in future studies.

It’s essential to acknowledge and discuss the limitations of this study. First, we examined only three out of numerous AM technologies. Additionally, we used a single machine and material from each technology due to practical constraints. While it’s not feasible to account for all possible variations, we aimed to include both affordable tabletop, as well as high-end, medically certified machines. The choice of materials aligned with our in-house standards for anatomical bone models. Second, the low number of participating surgeons (*n* = 6) limited our ability to conduct a more comprehensive statistical analysis of the questionnaire data, resulting in the presentation of data in a pooled manner for each question category.

## Conclusions

The results of this study, which evaluated the dimensional accuracy of bone models (0.08 ± 0.09 mm), considering three different technologies and various part orientations during the manufacturing process, highlight that AM models provide an accurate and precise representation of bone anatomy. However, when it comes to surgical simulation, FDM models received significantly lower ratings from the participating surgeons in terms of their anatomical and functional performance. On the other hand, PJ models were associated with notably higher costs. These findings emphasize the trade-offs and considerations that need to be made when choosing an additive manufacturing technology for anatomical bone models, particularly when they are intended for surgical simulation and training.

## Data Availability

The datasets generated and/or analysed during the current study are not publicly available due to ethical and legal reseans but are available from the corresponding author on reasonable request.
